# Development of the Bone Phenotype and microRNA Profile in Adults With Low‐Density Lipoprotein Receptor‐Related Protein 5–High Bone Mass (LRP5‐HBM) Disease

**DOI:** 10.1002/jbm4.10534

**Published:** 2021-09-01

**Authors:** Jens‐Jacob Lindegaard Lauterlein, Fatma Gossiel, Moritz Weigl, Richard Eastell, Matthias Hackl, Pernille Hermann, Jens Bollerslev, Morten Frost

**Affiliations:** ^1^ Department of Endocrinology and Metabolism Odense University Hospital Odense Denmark; ^2^ Department of Clinical Research University of Southern Denmark Odense Denmark; ^3^ Department of Oncology and Metabolism University of Sheffield Sheffield UK; ^4^ TAmiRNA GmbH Vienna Austria; ^5^ Department of Endocrinology Rikshospitalet Oslo Norway; ^6^ Faculty of Medicine University of Oslo Oslo Norway; ^7^ Steno Diabetes Centre Odense Odense University Hospital Odense Denmark

**Keywords:** HIGH BONE MASS, HR‐pQCT, *LRP5*, microRNA, RARE MONOGENETIC BONE DISEASE

## Abstract

Pathogenic variants in the Wnt‐pathway co‐receptor low‐density lipoprotein (LDL) receptor‐related protein 5 (LRP5) cause high bone mass (LRP5‐HBM) due to insensitivity to the endogenous antagonist of Wnt‐signaling. Although indicating incessant progression of BMD and biomarkers reflecting bone formation, this has not been confirmed in individuals with LRP5‐HBM. We investigated how the LRP5‐HBM bone phenotype changes with age in adults and is associated with quantitative changes of bone turnover markers and bone‐related microRNAs (miRNAs) in the circulation. Whole body, lumbar spine, total hip, and femoral neck areal BMD (aBMD) and radial and tibial bone microarchitecture and geometry were assessed using DXA and HR‐pQCT scans of 15 individuals with LRP5‐HBM_T253I_ (11 women; median age 51 years; range, 19 to 85 years) with a time interval between scans of 5.8 years (range, 4.9 to 7.6 years). Fasting P1NP and CTX were measured in 14 LRP5‐HBM_T253I_ individuals and age‐, sex‐, and body mass index (BMI)‐matched controls, and 187 preselected miRNAs were quantified using qPCR in 12 individuals and age‐, sex‐, and BMI‐matched controls. DXA and HR‐pQCT scans were assessed in subjects who had reached peak bone mass (aged >25 years, *n* = 12). Femoral neck aBMD decreased by 0.8%/year (*p* = 0.01) and total hip by 0.3%/year, and radial volumetric BMD (vBMD) increased 0.3%/year (*p* = 0.03). Differences in bone turnover markers at follow‐up were not observed. Compared to controls, 11 of the 178 detectable miRNAs were downregulated and none upregulated in LRP5‐HBM individuals, and five of the downregulated miRNAs are reported to be involved in Wnt‐signaling. Bone loss at the hip in LRP5‐HBM individuals demonstrates that the bone phenotype does not uniformly progress with age. Differentially expressed miRNAs may reflect changes in the regulation of bone turnover and balance in LRP5‐HBM individuals. © 2021 The Authors. *JBMR Plus* published by Wiley Periodicals LLC. on behalf of American Society for Bone and Mineral Research.

## 1 Introduction

Knowledge of the cell signaling pathways that regulate bone metabolism has emerged from studies of rare monogenetic bone disorders.^(^ ^1^ ^)^ The canonical Wnt pathway was linked to bone development and homeostasis in individuals with sclerosteosis due to a loss‐of‐function variant in *SOST* in 2001, which encodes sclerostin that acts as an endogenous inhibitor of Wnt signaling.^(^ ^2^, ^3^, ^4^ ^)^ Later, pathogenetic variants in the low‐density lipoprotein (LDL) receptor‐related protein 5 (*LRP5*), a Wnt co‐receptor, were linked with very high or low bone mass.^(^ ^5^, ^6^, ^7^ ^)^ These insights have contributed in the development of romosozumab, which is a sclerostin‐neutralizing human monoclonal antibody recently introduced as a treatment for osteoporosis.^(^ ^8^ ^)^

The Wnt signaling pathways are a group of cell‐signaling pathways of which canonical Wnt pathway refers to signal transduction through β‐catenin. Canonical Wnt‐signaling is initiated by binding of a Wnt‐ligand to a Frizzled receptor and the LRP5 co‐receptor leading to inactivation of the β‐catenin destruction complex.^(^ ^9^ ^)^ Subsequently, β‐catenin accumulates in the nucleus where it binds to the T‐cell factor (TCF) family of transcription factors and regulates the transcription of target genes, which promote osteoblastogenesis and suppress adipogenesis.^(^ ^10^ ^)^ Furthermore, Wnt‐signaling through LRP5 inhibits osteoclast differentiation.^(^ ^11^ ^)^

Pathogenic *LRP5* variants in the first beta propeller of LRP5^(^ ^12^ ^)^ may render the receptor insensitive to binding of sclerostin, an endogenous inhibitor. Thus the genetic variants is considered a gain‐of‐function because less inhibition by sclerostin cause activation of Wnt‐pathway causing a high bone mass phenotype known as LRP5‐HBM^(^ ^13^ ^)^ that may include otoneurological complications due to bone overgrowth,^(^ ^14^ ^)^ torus palatinus, and teeth encased in bone.^(^ ^15^ ^)^ Lumbar spine and forearm BMD increased with age in 19 LRP5‐HBM individuals with the T253I genetic variant,^(^ ^12^ ^)^ but not in a more recent study with 10 individuals carrying three different LRP5 variants.^(^ ^15^ ^)^ Although bone turnover markers indicated increased bone formation in four LRP5‐HBM individuals,^(^ ^7^ ^)^ decreased bone resorption and formation were observed in 19 individuals with LRP5‐HBM,^(^ ^12^ ^)^ and iliac crest bone biopsies display a reduced number and activity of osteoclasts supporting lower bone resorption in LRP5‐HBM individuals.^(^ ^16^ ^)^ Romosozumab, a sclerostin‐neutralizing drug, causes a transient uncoupling of bone formation and resorption,^(^ ^17^ ^)^ and homeostasis in bone turnover in individuals with LRP5‐HBM may resemble that observed in the later phases of romosozumab treatment with a lower level of resorption marker CTX. Because the anabolic effect declines after the first year of romosozumab treatment,^(^ ^18^, ^19^ ^)^ it is possible that bone mass accrual in a condition with partial sclerostin insensitivity such as LRP5‐HBM similarly subsides with age. However, prospective studies supporting continuous or cessation of bone mass accrual are missing.

Circulating microRNAs (miRNAs) are emerging as biomarkers that reflect disease status in osteoporosis.^(^ ^20^, ^21^, ^22^, ^23^ ^)^ miRNAs are small noncoding RNAs which exert posttranscriptional regulation of expression of several genes and can regulate osteoblast differentiation directly; eg, by targeting inhibitors of the Wnt co‐receptors LRP4/5/6^(^ ^24^, ^25^ ^)^ or the co‐receptor itself.^(^ ^26^ ^)^ Patients with severe osteoporosis due to a pathogenic variant in *WNT1*, a Wnt agonist, have normal levels of bone turnover markers^(^ ^27^ ^)^ but a distinct miRNA profile indicating disrupted feedback regulation of the Wnt pathway.^(^ ^28^ ^)^ Although Wnt1 is a LRP5 independent bone anabolic Wnt‐ligand,^(^ ^29^ ^)^ miRNAs could also display ongoing changes in the regulation of bone turnover in LRP5‐HBM.

Based on our previous cross‐sectional study in LRP5‐HBM_T253I_ individuals and controls that demonstrated associations between age and BMD but not bone turnover markers,^(^ ^12^ ^)^ we hypothesized that areal and volumetric BMD (aBMD and vBMD, respectively), microstructure, and geometry would increase with age in adult individuals with LRP5‐HBM, and that these changes would be reflected in circulating miRNAs involved in the promotion of bone formation.

## 2 Subjects and Methods

### 2.1 Study subjects

Nineteen LRP5‐HBM_T253I_ individuals from four different families sharing the same genetic variant in *LRP5* (T253I), previously participating in a clinical study of the LRP5‐HBM phenotype^(^ ^12^ ^)^ were invited by mail to participate in the present investigation. All LRP5‐HBM_T253I_ individuals replied, and 15 consented to participate. The medical history and results of a general physical examination were compared to their previous assessments. Bone scans were conducted in 15 participants, and blood samples were collected from 14 because one of the participants was pregnant at the time of sampling.

Fourteen healthy control subjects matched on age, sex, and body mass index (BMI) were recruited using public advertisement for assessments of bone turnover markers and miRNAs. Bone turnover and miRNA levels were assessed in 14 and 12 sets of cases and controls, respectively. The investigation was approved by the local ethics committee (file no. S20100113). All participants consented in writing and the study was performed in accordance with the Helsinki II declaration.

### 2.2 Anthropometrics

Body weight was measured with the participants wearing light clothing without shoes to the nearest 0.1 kg using a Seca model 708 scale (Seca, Hamburg, Germany) and height to the nearest 0.1 cm using a wall‐mounted Harpenden stadiometer (Holtain, Crymych, UK).

### 2.3 DXA and HR‐pQCT

Dual‐energy X‐ray absorptiometry (DXA) (Discovery A; Hologic, Waltham, MA, USA) was used to measure aBMD in the lumbar spine (L_1_–L_4_), total hip, femoral neck, and whole body. The coefficients of variation were 1.0% for all parameters.

A high‐resolution peripheral quantitative computed tomography (HR‐pQCT) system (XtremeCT; SCANCO Medical AG, Brüttisellen, Switzerland) was used to measure bone geometry, vBMD, and microarchitecture of the nondominant distal forearm and the distal part of tibia. The method has previously been validated and described in detail.^(^ ^30^, ^31^ ^)^ The manufacturer's default setting for patient scanning was applied, yielding a 9.02‐mm three‐dimensional (3D) representation of the radius/tibia in the axial direction. Coefficients of variation (CVs) for bone geometry, including cortical perimeter (Ct.perimeter), cortical area (Ct.Ar), and trabecular area (Tb.Ar), were 0.2% to 1.8%, whereas CVs of total bone vBMD, cortical vBMD (Ct.vBMD), and trabecular vBMD (Tb.vBMD) were 0.4% to 0.9%. CVs for microarchitectural parameters cortical thickness (Ct.Th), trabecular number (Tb.N), trabecular thickness (Tb.Th), and trabecular spacing (Tb.Sp) were in the range of 0.6% to 7.2%.

DXA and HR‐pQCT scans were performed in 2009 to 2010 and repeated in 2014 to 2017 on the same scanner.

### 2.4 Biological samples

After an overnight fast blood samples were drawn from the cubital vein. EDTA plasma were centrifuged at 1000*g* for 10 minutes at 4°C and serum were left for a maximum of 30 minutes at room temperature and centrifuged at 2100*g* for 10 minutes at 8°C. All sample were stored at −80°C within 1 hour and 15 minutes from sampling time. Frozen plasma and serum samples were shipped on dry ice.

### 2.5 Bone turnover markers

Serum samples for analyses of procollagen type 1 amino‐terminal propeptide (P1NP) and c‐telopeptide of type 1 collagen (CTX) were measured in 14 cases and controls at the University of Sheffield, UK, using an autoimmunoassay analyzer (Cobas e411; Roche Diagnostic, Mannheim, Germany) (CVs 1.5% and 4.4%, respectively).

### 2.6 miRNA analysis

Plasma levels of 187 circulating miRNAs related to bone metabolism were selected from previous studies^(^ ^20^, ^21^, ^32^ ^)^ and five quality controls were assessed in samples from 12 cases and controls using RT‐qPCR (LightCycler 480 platform) Total RNA was extracted from 200 μL plasma using the miRNeasy Mini Kit (Qiagen, Hilden, Germany). Samples were thawed on ice and centrifuged at 12,000*g* for 5 minutes to remove any cellular debris. For each sample, 200 μL of plasma were mixed with 1000 μL Qiazol and 1 μL of a mix of three synthetic spike‐in controls (Qiagen, Hilden, Germany). After a 10‐minute incubation at room temperature, 200 μL chloroform were added to the lysates followed by cooled centrifugation at 12,000*g* for 15 minutes at 4°C. Precisely 650 μL of the upper aqueous phase were mixed with 7 μL glycogen (50 mg/mL) to enhance precipitation. Samples were transferred to a miRNeasy mini column where RNA was precipitated with 750 μL ethanol followed by automated washing in a QiaCube liquid handling robot (Qiagen, Hilden, Germany) according to the manufacturer's recommendation. Finally, total RNA was eluted in 30 μL nuclease‐free water and stored at −80°C to await further analysis. Starting from total RNA samples, cDNA was synthesized using the miRCURY LNA RT kit (Qiagen, Hilden, Germany). Reaction conditions were set in accordance to the manufacturer's specifications. In total, 2 μL of total RNA were used per 10 μL reverse transcription (RT) reaction. PCR amplification was performed in a 384‐well plate format in a Roche LC480 II instrument (Roche Diagnostic) using miRCURY LNA SYBR Green PCR kit (Qiagen, Hilden Germany) with the following settings: 95°C for 10 minutes, 45 cycles of 95°C for 10 seconds, and 60°C for 60 seconds, followed by melting curve analysis. To calculate the cycle of quantification values (Cq‐values), the second derivative method was used. For quality control we added spike‐in controls UniSp2, UniSp4 and UniSp5 included in the RNA Spike‐In Kit (Qiagen, Hilden, Germany) prior to RNA extraction for estimation of the overall technical variance present in the raw data (Fig. S1 *A*). Cel‐miR‐39 was added to total RNA sample prior to reverse transcription and qPCR and UniSp3, a synthetic qPCR primer/DNA‐template mix was added on each qPCR plate to measure the technical variance of the qPCR reaction (Fig. S1 *A*). Hemolysis was assessed in all samples using the ratio of miR‐451a versus miR‐23a‐3p and applying a cut‐off of >5 for calling a sample hemolytic. Nine of the 187 preselected miRNAs had a low detection rate (Cq > 35) and/or more than four missing values, hence were not included in the analysis. Four control subjects were excluded as two had a hemolysis ratio >5 (Fig. S1 *B*) and two appeared as outliers in unsupervised principal component analysis (Fig. S2), causing heterogeneity and to reduce the risk of a type II error as the investigation was explorative. Thus, 12 LRP5‐HBM and eight control subjects were included in the final analyses of 178 miRNAs.^(^ ^33^ ^)^ Cq‐values of endogenous miRNAs were normalized to the RNA spike‐in control UniSp4 by subtracting the individual miRNA Cq‐value from the RNA spike‐in Cq‐value, thus obtaining delta‐Cq (dCq) values that were used for the analysis.

### 2.7 Statistics

Normality was assessed by histograms and qq‐plots and data are presented as mean (± standard deviation) for normal distribution or as median (interquartile range) for variables with a skewed distribution. Means were compared with unpaired *t* test between groups and paired *t* test within groups. Medians were compared with Wilcoxon rank sum test between groups and Wilcoxon signed rank test within groups. The change from scan #1 to scan #2 was tested by Wilcoxon signed‐rank test for rate of change being different from zero. Differential expression analysis of miRNA was performed using two‐sided unpaired *t* test. All statistical analyses were performed using Stata/IC release 15.1 (StataCorp, College Station, TX, USA). The bone‐related outcomes were considered complementary, and analyses of these outcomes were not corrected for multiple comparisons. For analysis of miRNAs, obtained *p* values were adjusted for multiple comparisons using the Benjamini Hochberg procedure. A false discovery rate (FDR) < 0.1 was considered as statistically significant. Statistical support was provided by Epidemiology, Biostatistics and Biodemography at the Department of Public Health, University of Southern Denmark.

## 3 Results

### 3.1 Participants–bone phenotype

A total of 15 LRP5‐HBM individuals participated in the investigation of the bone phenotype at baseline and time of follow‐up. None of the LRP5‐HBM individuals reported fractures between these time points. The medical history and physical examination did not reveal incident neurological complications; eg, visual impairment, dental complaints or clinically overt torus palatinus.

Studies of areal and volumetric bone density, geometry, and microarchitecture were performed with a median time interval of 5.8 years, ranging from 4.9 to 7.6 years for DXA and 5.8 to 7.6 years for HR‐pQCT. Median age at time of the first scan was 44.3 years (range, 13.0 to 79.2 years) and 50.5 years (range, 18.8 to 84.8 years) for the second scan for both DXA and HR‐pQCT scans. The three youngest subjects (one male and two females) were 13, 15, and 19 years when baseline DXA and HR‐pQCT scans were performed, and 19, 21, and 24 years as well as 19, 21, and 25 years at time of follow‐up to the DXA and HR‐pQCT scans, respectively. Large increments in BMD and substantial changes in bone microarchitecture were observed in the three youngest participants (Fig. S3), indicating that they had not reached peak bone mass. Therefore, to assess age‐related changes of the bone phenotype in adults, the three youngest individuals were excluded from further investigations. Anthropometrics for the 12 LRP5‐HBM individuals included in these analyses are presented in Table 1.

**Table 1:** Description of LRP5‐HBM Patients at Baseline and Time of Follow‐Up

Parameter	Baseline (*n* = 12)	Follow‐up (*n* = 12)
Age (years), median (range)	47.8 (23.6, 79.2)	53.7 (30.2, 84.8)
Sex (M/F), *n*	3/9	3/9
Height (cm), median (range)	170.4 (141.6, 181.9)	170.0 (140.0, 181.2)
Body weight (kg), median (range)	85.3 (65.0, 110.5)	87.5 (61.2, 108.5)
BMI (kg/m^2^), median (range)	30.8 (22.9, 36.6)	30.9 (20.5, 36.2)

### 3.2 Participants–miRNA measurements

A total of 12 cases and matched controls were included in cross‐sectional study of the miRNA measurements, and their anthropometrics are presented in Table 2.

**Table 2:** Description of LRP5‐HBM Patients and Controls for microRNA

Parameter	LRP5‐HBM (*n* = 12)	Controls (*n* = 12)	*p*
Age (years), median (range)	44.5 (18.5, 68.6)	41.5 (22, 69)	0.98
Sex (M/F), *n*	4/8	4/8	1
Height (cm), median (range)	174 (159, 182.5)	170.8 (162, 185.7)	0.79
Body weight (kg), median (range)	89.1 (66.8, 118.5)	91.5 (72.1,09.5)	0.98
BMI (kg/m^2^), median (range)	30.7 (21.4, 39.1)	31.4 (24.0, 39.3)	0.84

### 3.3 DXA

**Fig 1:**
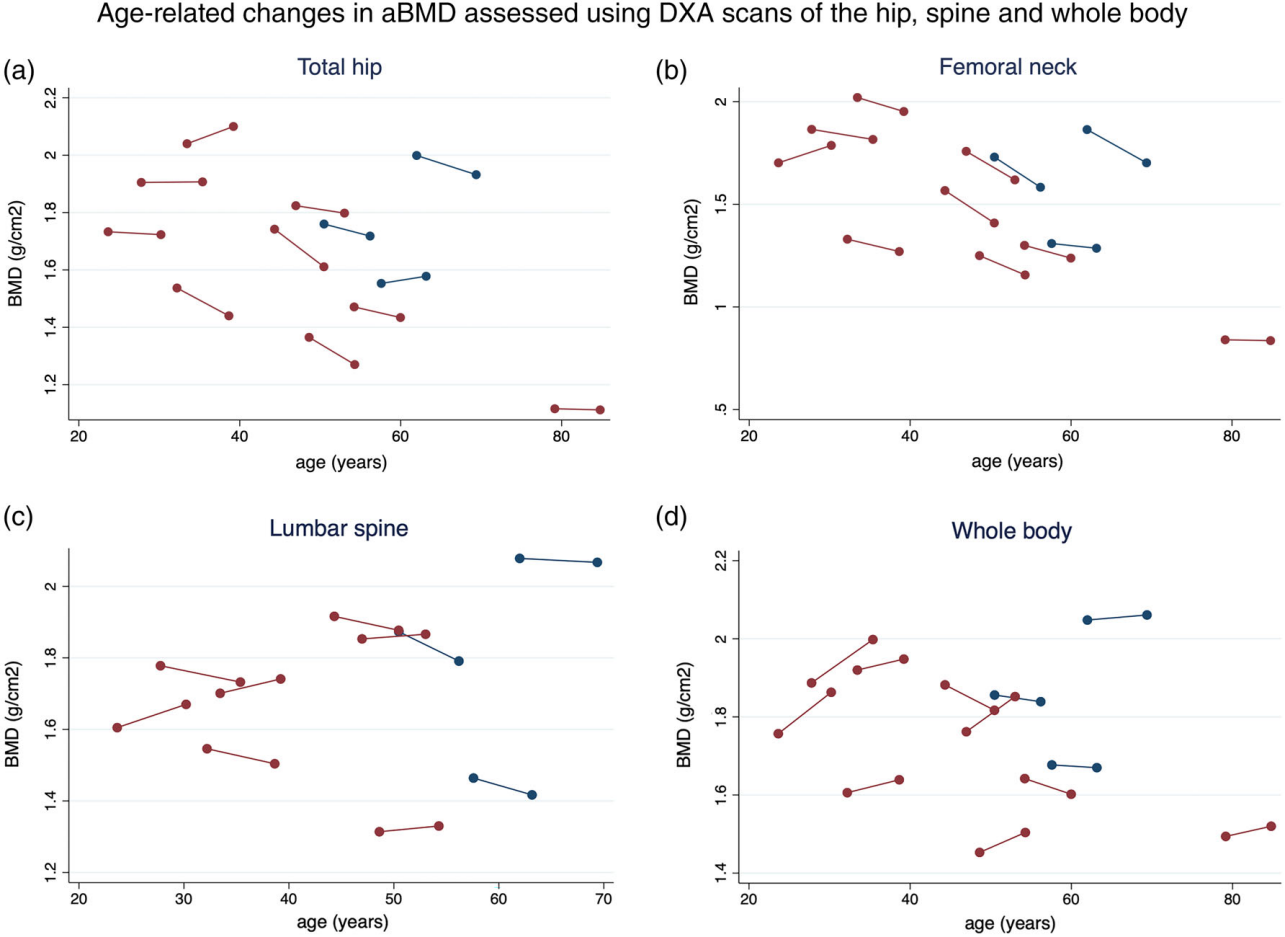
Demonstrates the change/D in BMD for each subjects between scan#1 and scan#2. Closed red circles: women. Closed blue circles: men.

Median aBMD in the 12 LRP5‐HBM individuals included in the analyses decreased both at the total hip (median [interquartile range]; from 1.74 g/cm^2^ [1.50, 1.87] to 1.67 g/cm^2^ [1.44, 1.85], *p* = 0.04, corresponding to an annual change of −0.33%) and femoral neck (from 1.63 g/cm^2^ [1.30, 1.81] to 1.50 g/cm^2^ [1.25, 1.75], *p* = 0.01, annual change: −0.76%) (Table 2, Fig. 1) which was not related to a change in bone area (data not presented). Assessment of lumbar spine aBMD was not possible in two subjects (aged 60.0 and 84.8 years) due to spinal stenosis and scoliosis. Changes in lumbar spine or whole‐body aBMD were not observed (Table 3, Fig. 1). At follow‐up, the median *Z*‐score was 6.0 (range, 3.3 to 9.6) at the hip and 5.7 (range, 3.3 to 9.7) at the lumbar spine.

**Table 3:** Measures of aBMD from DXA and vBMD From HR‐pQCT From the Baseline and Follow‐Up Scan

Parameter	Scan #1 (2009)	Scan #2 (2014–2017)	*p*	% Change/year	*p*
Areal BMD (DXA)					
Total hip (g/cm^2^)	1.74 (1.50, 1.87)	1.67 (1.44, 1.85)	0.04	−0.33 (−0.72, −0.02)	0.06
Femoral neck (g/cm^2^)	1.63 (1.30, 1.81)	1.50 (1.25, 1.75)	0.01	−0.76 (−1.32, −0.33)	0.01
Lumbar spine, (g/cm^2^). n = 10	1.74 (1.55, 1.87)	1.74 (1.50, 1.75)	0.38	−0.20 (−0.42, 0.22)	0.45
Total body (g/cm^2^)	1.76 (1.62, 1.88)	1.83 (1.62, 1.91)	0.14	0.28 (−0.12, 0.70)	0.12
vBMD (HR‐pQCT)					
Radius					
Total vBMD (mgHA/cm^3^)	541 (529, 594)	558 (525, 599)	0.81	−0.03 (−0.45, 0.78)	0.81
Cortical vBMD (mgHA/cm^3^)	893 (767, 930)	899 (869, 918)	0.81	−0.02 (−0.33, 0.31)	0.81
Trabecular vBMD (mgHA/cm^3^)	337 (288, 361)	338 (286, 374)	0.03	0.18 (−0.12,0.70)	0.03
Tibia					
Total vBMD (mgHA/cm^3^)	501 (462, 508)	495 (455, 513)	0.94	0.02 (−0.21, 0.22)	0.94
Cortical vBMD (mgHA/cm^3^)	881 (876, 923)	893 (880, 919)	0.69	0.06 (−0.14, 0.10)	0.64
Trabecular vBMD (mgHA/cm^3^)	313 (295, 345)	309 (288, 344)	0.43	−0.05 (−0.28, 0.10)	0.39

The annual change in %/year is calculated from the individual relative difference between the second and the first scan relative to the first scan and individual follow‐up time. BMD and annual change in %/year are presented as median (interquartile range).

### 3.4 HR‐pQCT

**Fig 2:**
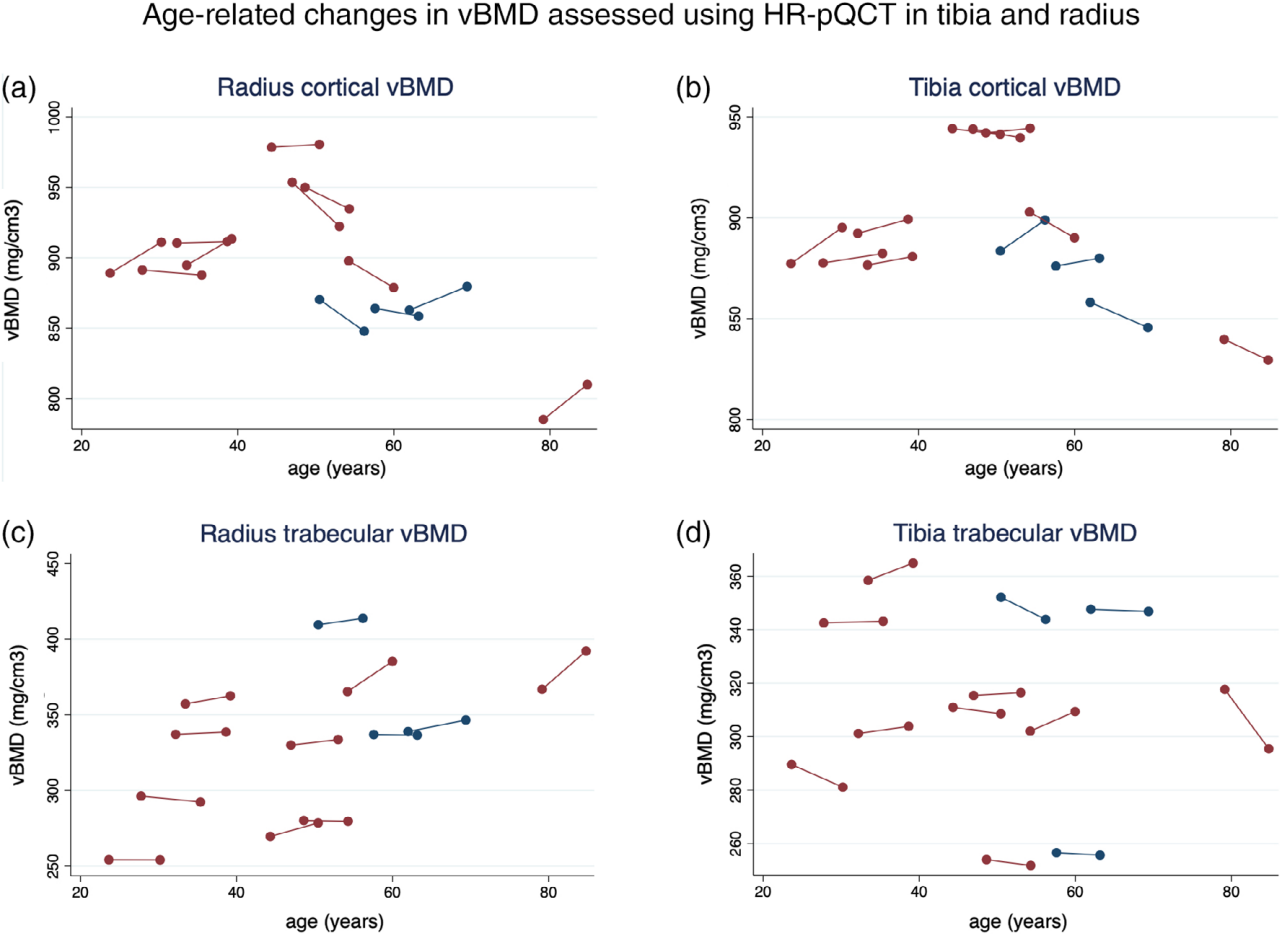
Demonstrates the change/D in vBMD in radius and tibia for each subjects between scan#1 and scan#2. Closed red circles: women. Closed blue circles: men.

Trabecular vBMD increased in radius (from 337 mg hydroxyapatite (HA)/cm^3^ [288, 361] to 338 mgHA/cm^3^ [286, 374], *p* = 0.03) by 0.18% per year but not in tibia (Table 3, Fig. 2). Although the ratio of the trabecular bone volume over the total cancellous tissue volume (BV/TV) increased (0.281 [0.241, 0.301] to 0.282 [0.229, 0.312], *p* = 0.03) by 0.19% per year and trabecular network distribution inhomogeneity (SD.1/Tb.N) decreased (0.127 mm [0.109, 0.161] to 0.122 mm [0.109, 0.147], *p* = 0.05) by 0.77% per year in radius, other measures of bone geometry and microarchitecture such as trabecular thickness and number, cortical area, and bone perimeter were unchanged (Table 4).

**Table 4:** Geometry and Microarchitecture Assessed by HR‐pQCT From the Baseline and Follow‐Up Scan

Parameter	Scan #1 (2009)	Scan #2 (2014–2017)	*p*	% Change/year	*p*
Radius					
Bone area					
Perimeter (mm)	71.3 (68.1, 78.7)	72.6 (69.1, 79.5)	0.24	0.16 (−0.01, 0.6)	0.18
Cortical bone area (mm^2^)	124.1 (102.8, 164.7)	123.9 (102.7, 177.9)	0.16	0.39 (−0.38, 1.04)	0.14
Trabecular bone area (mm^2^)	188.5 (160.2, 217.6)	198.6 (159.7, 223.2)	0.58	0.28 (−1.00, 1.27)	0.58
Cortical parameters					
Cortical thickness (mm)	1.71 (1.54, 2.11)	1.72 (1.49, 2.29)	0.97	−0.29 (−0.73, 0.94)	0.87
Trabecular parameters					
Bone volume/tissue volume (ratio)	0.281 (0.241, 0.301)	0.282 (0.229, 0.312)	0.03	0.19 (0, 0.40)	0.03
Trabecular number (1/mm)	2.4 (2.1, 2.5)	2.3 (2.2, 2.5)	0.14	0.50 (−0.22, 1.07)	0.12
Trabecular thickness (mm)	0.12 (0.12, 0.13)	0.12 (0.11, 0.13)	0.91	0.12 (−0.73, 0.65)	0.94
Trabecular spacing (mm)	0.32 (0.29, 0.35)	0.30 (0.29, 0.33)	0.10	−0.80 (−1.14, 0.13)	0.10
SD.1/Tb.N (mm)	0.127 (0.109, 0.161)	0.122 (0.109, 0.147)	0.05	−0.77 (−1.90, 0.08)	0.06
Tibia					
Bone area					
Perimeter (mm)	114.2 (103.9, 119.4)	113.3 (103.6, 118.7)	0.11	−0.06 (−0.14, 0.01)	0.07
Cortical bone area (mm^2^)	242.2 (211.6, 308.8)	241.1 (213.5, 310.1)	0.81	0.01 (−0.23, 0.17)	0.81
Trabecular bone area (mm^2^)	567.4 (492.8, 642.8)	565.6 (486.0, 637.1)	0.14	−0.19 (−0.47, 0.05)	0.12
Cortical parameters					
Cortical thickness (mm)	2.33 (1.97, 2.64)	2.33 (1.92, 2.69)	1.00	0.00 (−0.30, 0.21)	0.87
Trabecular parameters					
Bone volume/tissue volume (ratio)	0.261 (0.246, 0.288)	0.258 (0.240, 0.287)	0.35	−0.07 (−0.26, 0.09)	0.33
Trabecular number (1/mm)	2.6 (2.4, 2.8)	2.6 (2.5, 2.6)	0.56	−0.12 (−0.66, 0.34)	0.56
Trabecular thickness (mm)	0.10 (0.10, 0.11)	0.10 (0.10, 0.11)	0.84	0.0 (−0.61, 0.45)	0.72
Trabecular spacing (mm)	0.29 (0.26, 0.31)	0.29 (0.28, 0.31)	0.50	0.13 (−0.24, 0.72)	0.05
SD.1/Tb.N (mm)	0.115 (0.099, 0.120)	0.116 (0.104, 0.121)	0.35	0.28 (−0.23, 1.31)	0.31

The annual change in %/year is calculated from the individual relative difference between the second and the first scan relative to the first scan and individual follow‐up time. HR‐pQCT parameters and annual change in %/year are presented as median (interquartile range). SD.1/Tb.N = trabecular network inhomogeneity (standard deviation of 1/Tb.N).

### 3.5 Bone turnover markers

Fasting bone turnover markers were measured in 14 subjects and controls at time of follow‐up. Levels of P1NP and CTX did not differ between LRP5‐HBM and controls (median [interquartile range]; P1NP (ng/mL): 60.5 [37.9, 93.7] versus 62.7 [40.2, 76.0], *p* = 0.55; and CTX (ng/mL): 0.37 [0.31, 0.61] versus 0.43 [0.28, 0.52], *p* = 0.91).

### 3.6 miRNA

**Fig 3:**
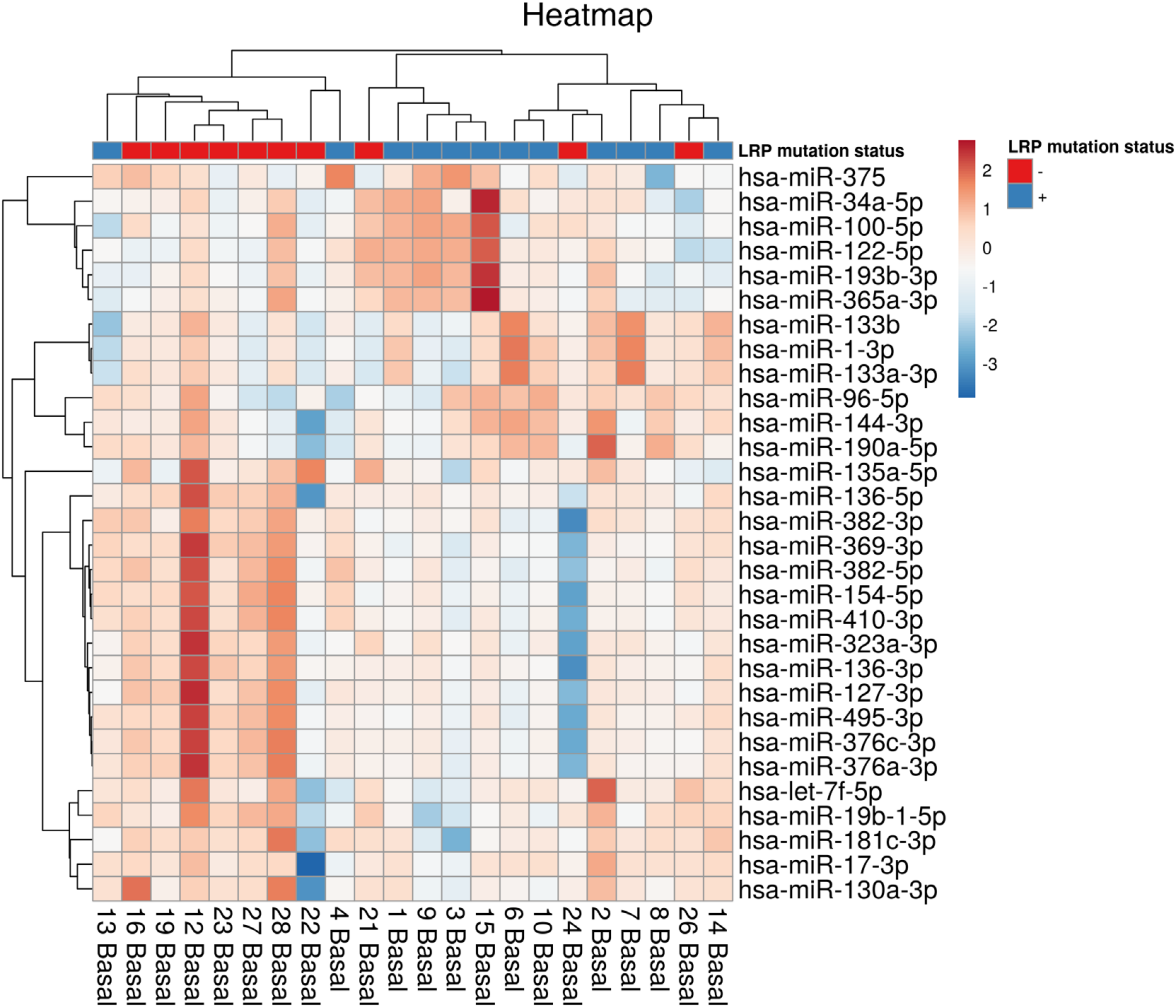
Spike‐in normalized dCq values for the Top 30 microRNAs (ranked according to their coefficient of variation) were used to draw a heatmap. Rows represent microRNA, and columns represent samples. Pearson correlation and complete linkage were used for clustering of samples.

**Fig 4:**
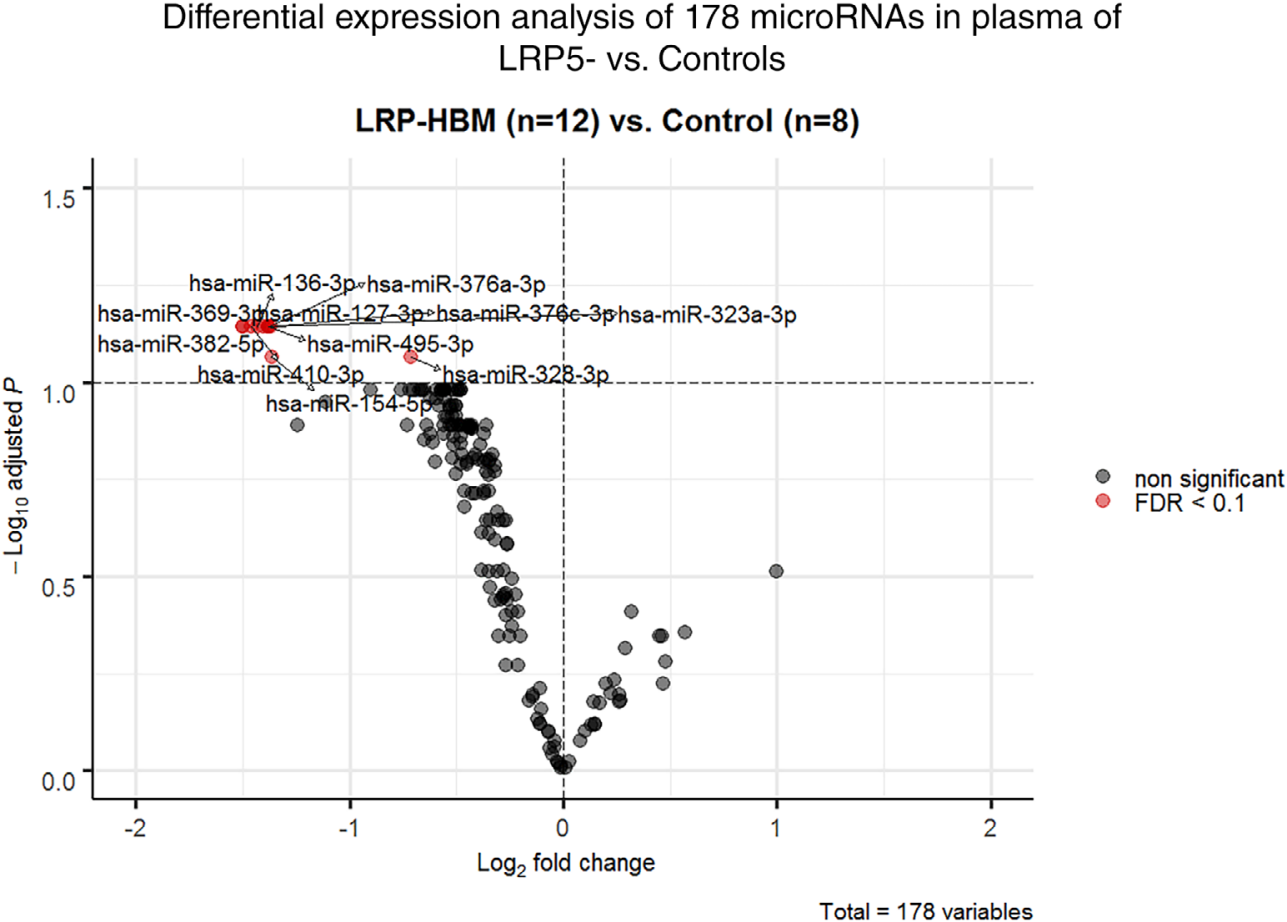
Scatterplots for 11 significantly regulated miRNAs (FDR<0.1) in 12 mutation‐positive subjects (LRP+) heterozygous for the T253I genetic variant in LRP5 and in 8 mutation‐negative subjects (LRP−). Spike‐in normalized delta Cq values are shown with mean and standard deviation indicated.

**Fig 5:**
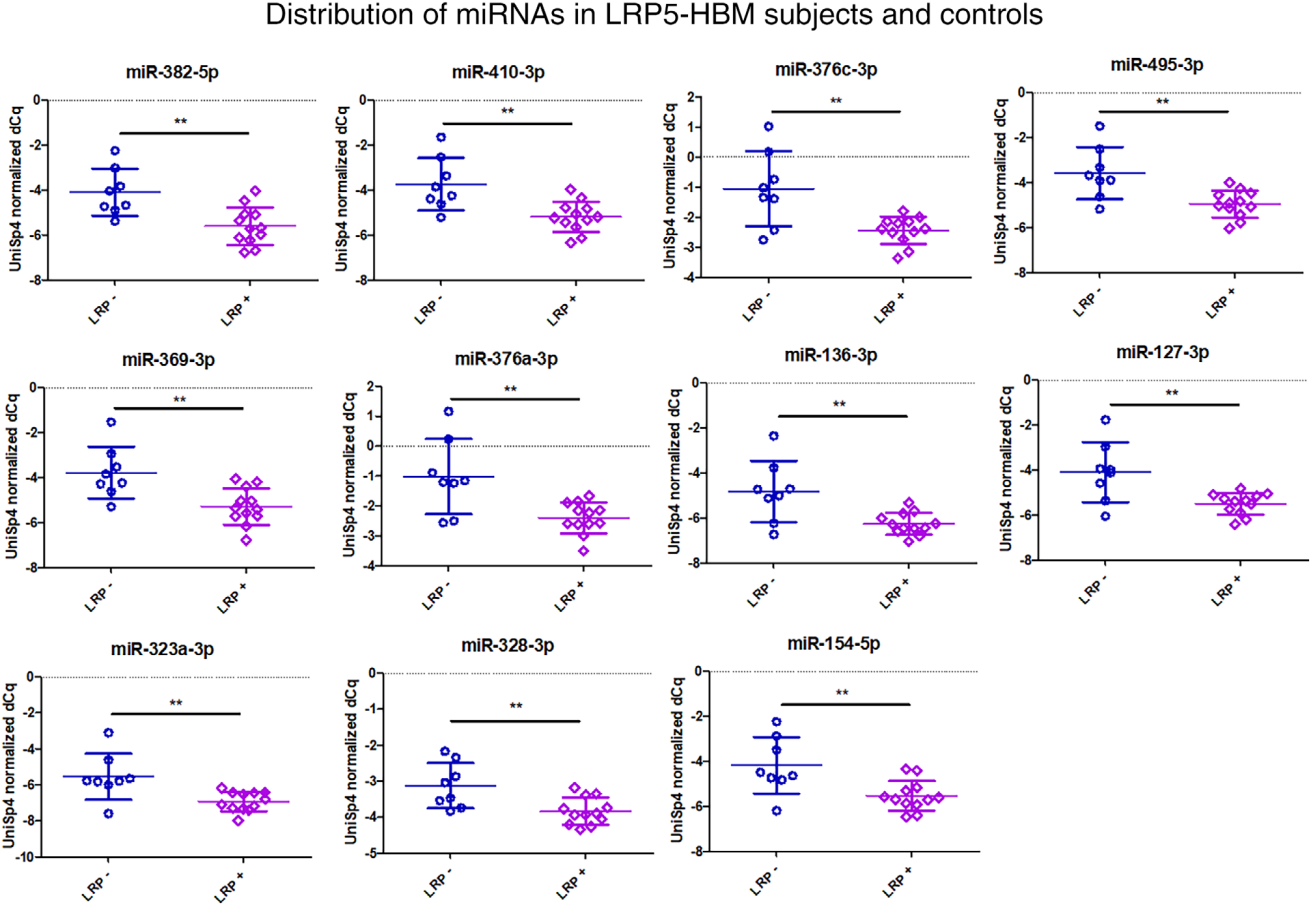
Scatterplots for 11 significantly regulated miRNAs (FDR<0.1) in 12 mutation‐positive subjects (LRP−) heterozygous for the T253I genetic variant in LRP5 and in 8 mutation‐negative subjects (LRP−). Spike‐in normalized delta Cq values are shown with mean and standard deviation indicated.

**Fig 6:**
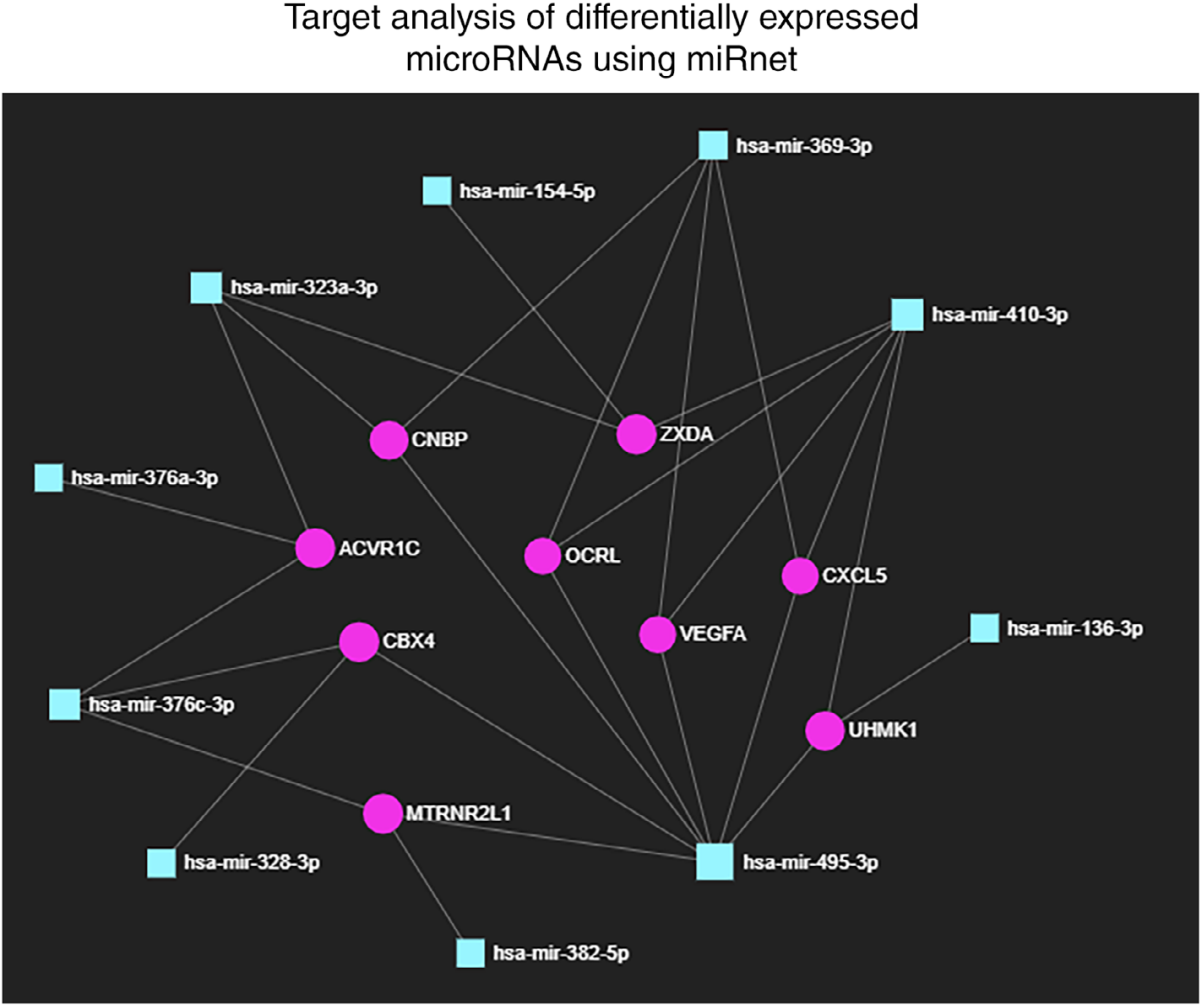
Eleven microRNAs differentially regulated with a FDR < 0.1 in LRP5‐HBMT253I vs. Controls were used for constructing a target network using the online tool miRnet (accessible via mirnet.ca). The degree filter for constructing the network was set to 2, hence only target nodes with at least two connections remained in the network.

Among the 30 most variant miRNAs based on CV%, a distinct clustering of LRP5‐HBM subjects was observed (Fig. 3) and subsequent differential expression analysis revealed a significant downregulation of 11 miRNAs (Figs. 4 and 5: miR‐369‐3p, miR‐495‐3p, miR‐323a‐3p, miR‐410‐3p, miR‐382‐5p, miR‐376c‐3p, miR‐376a‐3p, miR‐136‐3p, miR‐154‐5p, miR‐328‐3p, and miR‐127‐3p. All FDR < 0.1). Five of these have been demonstrated to interact with components of the Wnt‐pathway: miR‐410‐3p, miR‐376c‐3p, miR‐136‐3p, miR‐154‐5p, and miR‐328‐3p.^(^ ^34^, ^35^, ^36^, ^37^, ^38^ ^)^ The web‐based service https://www.mirnet.ca/ was used to build a network of experimentally verified target genes to identify common targets of the 11 differently expressed miRNAs. Only miRNAs with at least one common target with another miRNA were allowed to stay in the network, using this criterion all but mir‐127‐3p remained for constructing the network (Fig. 6). The network analyses revealed nine genes, including *ZXDA*, *CNBP*, *OCRL*, *ACVR1C*, *VEGFA*, *UHMK1*, *CBX4*, *CXCL5*, and *MTRNR2L1*. Of these, *VEGFA*, *UHMK1*, and *CXCL5* are known to relate directly to bone development or homeostasis, and *CNBP* is known to modulate Wnt‐signaling directly in zebrafish.^(^ ^39^ ^)^ The remaining genes have no currently apparent association with bone.

## 4 Discussion

This study demonstrated that areal and volumetric bone density, microarchitecture, and geometry remained stable during a 6‐year follow‐up study of adult individuals with LRP5‐HBM_T253I_. We observed a loss of aBMD at the femoral neck and total hip and an increase in trabecular vBMD at the radius as well as a change in bone microarchitecture with an increase in trabecular bone volume fraction in radius. Although assessments of biochemical markers did not indicate differential bone turnover in LRP5‐HBM_T253I_ as compared to closely matched controls, several bone‐related miRNAs were downregulated in LRP5‐HBM_T253I_ participants.

### 4.1 DXA and HRpQCT

Prospective DXA data indicated bone loss at the hip but not spine, and the annual decrease in the femoral neck and hip BMD was similar to or even slightly greater than the 0.35% to 0.55%/year that is observed in healthy Danish men and premenopausal and postmenopausal women,^(^ ^40^ ^)^ further supporting that individuals with LRP5_T253I_‐HBM are not protected from age‐related bone loss at least at the hip and femoral neck. The impact of the pathogenic variant could be compartment specific as the hip mostly consists of cortical bone whereas the spine is dominated by trabecular bone.^(^ ^41^ ^)^ Importantly, spinal degenerative changes including osteoarthritis are commonly observed in patients with high bone mass^(^ ^42^ ^)^ and may falsely increase BMD. Although LRP5‐HBM patients with overt spinal diseases were excluded from the analyses, degenerative changes may have masked age‐related spinal bone loss in individuals with LRP5‐HBM. Increases in trabecular variables were observed in the radius only and the increase in trabecular vBMD in radius was in accordance with a population‐based study of Danish men and women.^(^ ^43^ ^)^ Changes in trabecular thickness and number, cortical parameters, and bone areas including perimeter were not apparent at any of the peripheral sites. These findings differ from the Danish population‐based study^(^ ^43^ ^)^ showing age‐related decreases in trabecular number (postmenopausal women) and thickness (men 20 to 49 years) in radius only,^(^ ^43^ ^)^ indicating that the LRP5 variant prevents deterioration of trabecular bone microarchitecture and possibly increases trabecular BMD in radius. The absence of changes in individuals with LRP5‐HBM in some of the HR‐pQCT–derived measures are in line with the prospective data in healthy men >50 years regarding; eg, cortical area or trabecular number in radius^(^ ^43^ ^)^; however, these findings are at odds with data in postmenopausal women (decreasing) and younger men and women (increasing),^(^ ^43^ ^)^ showing that the heterogeneity of our cohort with regard to sex, age, and menopausal status makes comparisons with these prospective data challenging. Jointly, our findings indicate that the LRP5 variant may prevent bone loss in radius but does not cause a continuous and general increase in bone mass, microarchitecture, or geometry.

Contrary to our previous publication that demonstrated lower CTX but normal P1NP levels in LRP5‐HBM cases,^(^ ^12^ ^)^ differences in bone turnover markers between cases and controls were not observed in the present investigation. Different study populations may explain this as the present investigation included fewer and older LRP5‐HBM individuals. BMI of the LRP5‐HBM patients was approximately 30 kg/m^2^, and increases in BMI generally associate with lower levels of bone turnover markers. Therefore, similar levels of bone turnover markers in the present investigation may be explained by inclusion of BMI‐matched controls, which was not done in the previous study.

### 4.2 miRNA

We identified 11 miRNAs that were downregulated including five reported to interact directly with components of the Wnt‐pathway.^(^ ^34^, ^35^, ^36^, ^37^, ^38^ ^)^ Among these, repression of miR‐328‐3p impairs osteogenic differentiation in human mesenchymal stem cells^(^ ^32^ ^)^ as downregulation of miR‐328‐3p promotes Axin1 activity, which inhibits Wnt‐signaling.^(^ ^37^ ^)^ miR‐154‐5p is associated with osteogenic differentiation^(^ ^44^ ^)^ due to suppression of *DKK2*, a Wnt antagonist,^(^ ^34^ ^)^ and lower miR‐154‐5p indicates increased translation of *DKK2* and subsequently inhibition of Wnt‐signaling. Finally, we observed lower levels of miR‐410‐3p, reported to repress the Wnt antagonist *DKK1* in colorectal cancer,^(^ ^45^ ^)^ as well as *BMP2*, which induces osteogenesis.^(^ ^45^ ^)^ Downregulation of these three miRNAs may upregulate Wnt antagonists thus impairing Wnt signaling and suppressing osteogenic differentiation and osteoblast activity. miR‐410‐3p^(^ ^36^ ^)^ and miR‐376c‐3p^(^ ^35^ ^)^ inhibit expression of Wnt3a, which promotes Wnt‐signaling. Although downregulation of these miRNAs may promote Wnt signaling, the overall outcomes of the contrasting effects of miR‐410‐3p on Wnt signaling remain unknown. We observed downregulation of miR‐136‐3p, which inhibits osteoblast differentiation and promotes osteoclastogenesis,^(^ ^46^ ^)^ possibly by repressing the Wnt agonist *WNT2*.^(^ ^38^ ^)^ Thus, upregulation of Wnt agonists and BMP‐signaling by downregulation of miR‐376c‐3p, miR‐410‐3p, and miR‐136‐3p could promote bone formation and increase bone mass. It is possible that downregulation of miRNAs controlling Wnt‐antagonists represent a regulatory mechanism that counteracts unbalanced bone formation and resorption in individuals with LRP5‐HBM, possibly contributing to an increase in serum sclerostin as observed in patients with this condition.^(^ ^12^ ^)^ Downregulation of miR‐136‐3p is also reported to inhibit differentiation of osteoclasts,^(^ ^46^ ^)^ which could explain the reduced osteoclast number and activity observed in bone biopsies from LRP5‐HBM patients.^(^ ^16^ ^)^ We speculate that differential expression of miRNAs in LRP5‐HBM individuals reflect the disease mechanism as well as counter‐regulatory responses.

Assessments of patients with WNT1 osteoporosis, another rare, Wnt‐pathway‐related bone disease, revealed upregulation of two miRNAs and downregulation of six miRNAs.^(^ ^28^ ^)^ Neither of the miRNAs differentially expressed in WNT1 osteoporosis overlapped with the miRNA profile identified in LRP5‐HBM individuals, possibly due to the bone anabolic effect of WNT1 being independent of LRP5.^(^ ^29^ ^)^ Similarly, we did not observe overlap with miRNAs that were differentially expressed in osteoporotic patients with vertebral fractures.^(^ ^22^ ^)^

Of the nine genes identified in the network analysis, *VEGFA*, *UHMK1*, and *CXCL5* are known to be directly involved in bone development or homeostasis. In addition, *CNBP* modulates Wnt‐signaling during embryonic mesenchymal differentiation in zebrafish,^(^ ^39^ ^)^ and *ACVR1C* ^(^ ^47^ ^)^ and *ZXDA* ^(^ ^48^ ^)^ interact with Wnt‐signaling, but the effect on bone cells or their precursors are unknown. Importantly, our findings need to be corroborated in other LRP5‐HBM individuals and animal models, and further studies of the miRNAs identified in the LRP5‐HBM individuals are needed to establish their effects on human bone cells.

The differentially expressed miRNAs identified may have different effects on bone accrual and after peak bone mass has been reached. Although the present study included a relatively large number of LRP5‐HBM cases, an imbalance in the number of men and women in the investigation precluded an assessment of sex‐specific effects of the genetic variant. Future clinical and mechanistic investigations; eg, in animal LRP5 HBM models, may clarify if the effects of the gene variants depend on the sex, including menopausal status. Additionally, spurious associations may have emerged in the miRNA analyses because controls were not matched for ancestry. It should be acknowledged that the present investigation was restricted to the characterization of a single genetic variant, and the results may differ in carriers of other *LRP5* variants.

## 5 Conclusion

This study demonstrates that the LRP5‐HBM condition does not progress in adults. Contrary to the general population, bone density, microstructure, and geometry remain stable at most of the sites investigated. Although LRP5‐HBM individuals are not entirely protected from age‐related bone loss, indications of clinically relevant changes in fracture risk were not observed. The downregulation of several miRNAs in individuals with the T253I gain‐of‐function genetic variant in *LRP5* predicted to reduce sclerostin sensitivity may reflect a counter‐regulatory mechanism maintaining homeostasis as reflected by normal bone turnover markers.

## Conflict of Interest

MH and MW report personal fees from TAmiRNA GmbH, outside the submitted work. RE reports grants from Amgen, grants and personal fees from IDS, grants from Alexion, grants and personal fees from Roche, personal fees from GSK Nutrition, personal fees from Mereo, personal fees from Sandoz, grants and personal fees from Nittobo, personal fees from AbbVie, personal fees from Samsung, personal fees from Haoma Medica, personal fees from Elsevier, personal fees from CL Bio, personal fees from FNIH, personal fees from Viking, personal fees from UCSF, personal fees from Biocon, from Lyramid, outside the submitted work.

## Supporting information

**Figure S1 Quality control of circulating microRNA analysis in plasma. (***A*) Raw Cq‐values obtained for RNA spike‐in controls (UniSp2, 4, and 5), cDNA spike‐in controls (cel‐miR‐39‐3p), and qPCR controls (UniSp3) are plotted for all samples. (*B*) Hemolysis was monitored for each sample using the ratio of miR‐23a‐3p versus miR‐451a‐5p. The ratio threshold of 5 for calling a sample hemolytic is indicated as orange line. Two samples crossing this threshold were excluded from the analysis.
Figure S2. Principal component analysis of miRNA in plasma
Figure S3. Changes in vBMD in all participants and in those aged at least 25 years
