# Supplementary material for: Development of the Bone Phenotype and microRNA Profile in Adults With Low‐Density Lipoprotein Receptor‐Related Protein 5–High Bone Mass (LRP5‐HBM) Disease
Source: JBMR Plus. 2021 Sep 1;5(9):e10534. doi: 10.1002/jbm4.10534 (PMC8441296; doi:10.1002/jbm4.10534)
Supplement: Supplementary file 1 — Figure S1 Quality control of circulating microRNA analysis in plasma. (A) Raw Cq‐values obtained for RNA spike‐in controls (UniSp2, 4, and 5), cDNA spike‐in controls (cel‐miR‐39‐3p), and qPCR controls (UniSp3) are plotted for all samples. (B) Hemolysis was monitored for each sample using the ratio of miR‐23a‐3p versus miR‐451a‐5p. The ratio threshold of 5 for calling a sample hemolytic is indicated as orange line. Two samples crossing this threshold were excluded from the analysis. Figure S2. Principal component analysis of miRNA in plasma Figure S3. Changes in vBMD in all participants and in those aged at least 25 years [file JBM4-5-e10534-s001.doc]

**Supplemental figures**

**Figure S1**

**Quality control of circulating microRNA analysis in plasma.**

A) Raw Cq-values obtained for RNA spike-in controls (UniSp2, 4, and 5), cDNA spike-in controls (cel-miR-39-3p), and qPCR controls (UniSp3) are plotted for all samples. B) Hemolysis was monitored for each sample using the ratio of miR-23a-3p versus miR-451a-5p. The ratio threshold of 5 for calling a sample hemolytic is indicated as orange line. Two samples crossing this threshold were excluded from the analysis.

**A)**

**
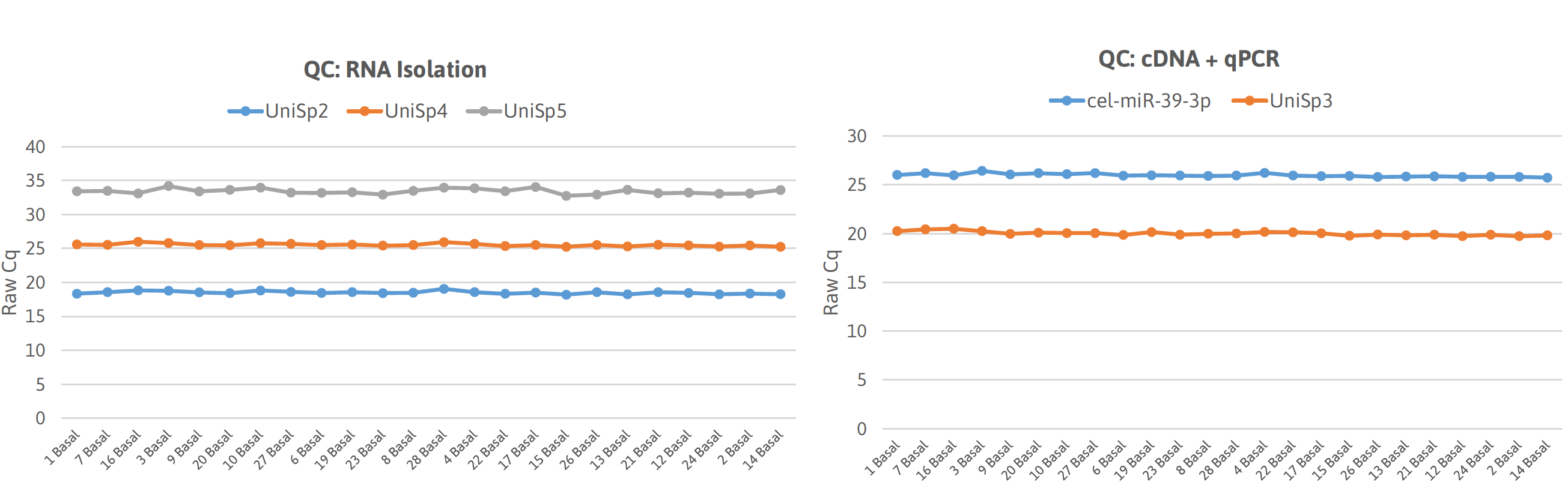
**

**B)**

**
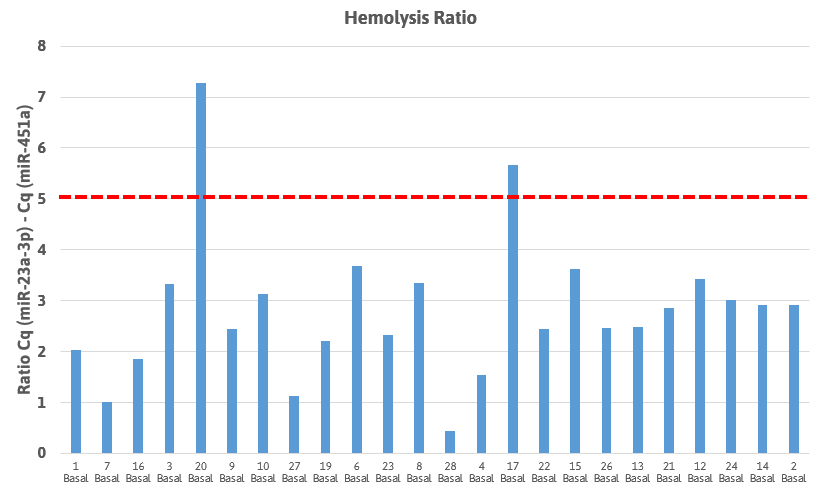
**

**Figure S2. Principal component analysis of miRNA in plasma**


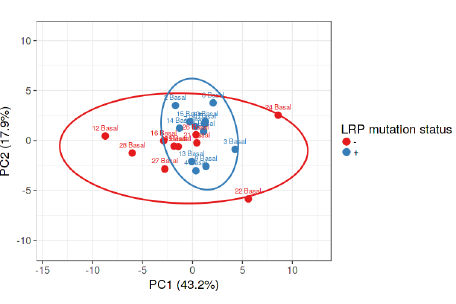


Principal Component Analysis (PCA) plot showing clustering of patients based on their plasma microRNA expression levels of the 30 most variant microRNAs (according to CV%). Control patients are depicted in red and LRP5-HBM_T253I_ are shown in blue. Two control subjects had markedly different microRNA profiles, and data from these individuals were removed from the analysis. Two-dimension coordinates were plotted for 12 carriers of the genetic variant (blue) and healthy controls (red). X-axis shows principal component 1 that explains 43.2% of the total variance. Y-axis shows principal component 2 that explains 17.0% of the total variance

**Figure S3. Changes in vBMD in all participants and in those aged at least 25 years**

Tibia cortical vBMD including all 15 subjects (left) and only those above 25.2 years of follow-up, who were considered to have reached peak bone mass.
